# Effects of surgery, chemotherapy, and radiation on hepatocellular carcinoma patients: A SEER-based study

**DOI:** 10.1016/j.amsu.2021.102782

**Published:** 2021-09-04

**Authors:** Kaif Qayum, Irfan Kar, Usman Rashid, Ghulam Nawaz, Praveena Krishnakumar, Veena Sudarshan, Aliraza Syed

**Affiliations:** a Department of General Surgery, Hereford County Hospital, Wye Valley NHS Trust, Hereford, Herefordshire, UK; b Department of Medicine, Hereford County Hospital, Wye Valley NHS Trust, Hereford, Herefordshire, UK; c University of Buckingham Medical School, Buckingham, Buckinghamshire, UK

**Keywords:** Hepatocellular carcinoma, SEER, Surgery, Chemotherapy, Radiotherapy, Mortality

## Abstract

**Background:**

Hepatocellular carcinoma (HCC) is a major global health issue, accounting for 75%–85% of primary liver cancer cases. HCC has huge molecular heterogeneity, and the treatment varies among the patients. The aim of this study is assess the effect of surgery, chemotherapy, and radiation on the mortality risk in hepatocellular carcinoma (HCC) patients.

**Methods:**

A retrospective cohort study, obtaining HCC patients' data from the Surveillance, Epidemiology, and End Results (SEER) database. The analyses were conducted using the SPSS software. We investigated the effect of surgery, chemotherapy, and radiation on the mortality risk factors using the Kaplan–Meier and the Cox regression tests in the univariate and multivariate analyses.

**Results:**

A total of 68270 HCC patients, of whom 56347 patients died, were analyzed. In patients who performed surgery, the mortality risk was higher in patients aged ≥50 years, Black, single and widowed, regional and distant stages, and grades II, III, and IV (HR, 1.143), (HR, 1.057), (HR, 1.095), (HR, 1.284), (HR, 1.341), (HR, 2.291), (HR, 1.125), (HR, 1.711), and (HR, 1.894) respectively. In patients who received chemotherapy, the risk was lower in females (HR, 0.948), but higher in widowed (HR, 1.143), in regional and distant stages (HR, 1.479), and (HR, 2.439) respectively, and grades III, and IV (HR, 1.741), and (HR, 1.688) respectively. In patients who received beam radiation, the risk was higher in Black (HR, 1.195), widowed (HR, 1.181), regional (HR, 1.439), and distant stages (HR, 2.287), and in grades III (HR, 1.594), and IV (HR, 1.694).

**Conclusion:**

In HCC patients, Black, widowed, regional, and distant stages, grades III and IV had higher mortality risks in several treatment options. In patients who underwent surgery, ≥50 years and grade II also had a higher risk. We recommend future research to assess the radiation sequence with surgery.

## Highlights

• We included 68270 hepatocellular carcinoma patients, of whom 56347 patients died.
• We assessed the effect of surgery, chemotherapy, and radiation in HCC patients.
• Patients underwent surgery, aged ≥50 years and grade II had higher mortality risks.
• Regional and distant stages, grades III and IV had higher mortality with all groups.
• Females had lower mortality risk with all groups except patients received radiation.

## 1 Introduction

Hepatocellular carcinoma (HCC) is a common global malignancy with considerable morbidity and mortality [1,2]. HCC is responsible for 75%–85% of primary liver cancer cases, and primary liver cancer is globally the sixth most commonly diagnosed cancer and the third leading cause of cancer death [3].

Various sociodemographic features were associated with HCC, mainly in patients with cirrhosis [4]. For example, the incidence increases with advancing age in all populations [5]. Moreover, both incidence and mortality rates are 2–3 times higher among males in most world regions. This is possibly due to a clustering of risk factors among males and the differences in the sex hormones [4,6].

Very early or early stage HCC (BCLC 0, A) patients are eligible for curative surgical treatment and locoregional ablation, yielding survival times of >5 years. Intermediate stage (BCLC B) patients receive transarterial chemoembolization with <2–5 survival years. Radiation therapy can be used solely or as a crucial element of combined therapy [7]. Unfortunately, the prognosis of HCC is poor worldwide [8].

HCC is known for its huge molecular heterogeneity [9]. Also, the treatment options differ among patients and greatly depend on the disease stage [10]. Therefore, it is important to study the effect of different factors according to the treatment. So, this study aims to assess the impact of several variables regarding different treatment options (surgery, chemotherapy, radiation, and radiation sequence with surgery) on the mortality risk in patients with HCC.

## 2 Methods

### 2.1 Study design

We followed the “Strengthening the Reporting of Observational Studies in Epidemiology (STROBE)" guidelines to conduct this retrospective cohort study [11]. We also followed the Strengthening The Reporting Of Cohort Studies in Surgery (STROCSS) criteria [12]. We registered the study protocol in Research Registry (UIN: researchregistry7087) [13].

### 2.2 Ethical approval

SEER data are publicly available and anonymized. So, the ethical approval was waived.

### 2.3 Study population and data collection

Our sample included all available patients’ data diagnosed with hepatocellular carcinoma registered in the Surveillance, Epidemiology, and End Results (SEER) database from 1975 to 2016. SEER database involves data of cancer patients in the United States and makes the data accessible upon request. We grouped patients according to the treatment received; patients with or without surgery, patients with or without chemotherapy, and patients without radiotherapy. The follow-up period was until the end of 2016 or until death. We collected data concerning age, sex, marital status (single, married, divorced, separated, and widowed), race (White, Black, and others (American Indian/AK Native, Asian/Pacific Islander)), stage of the disease (localized, regional, and distant), grade (Grade I; well-differentiated, Grade II moderately differentiated, Grade III; poorly differentiated, Grade IV; undifferentiated; anaplastic, and unknown), chemotherapy (yes or no), surgery (yes or no), radiation (beam, others, and none), radiation sequence with surgery (after surgery, intraoperative, prior to surgery, no radiation or surgery, radiation before and after, and surgery before and after). Our primary outcomes are to identify survival months and mortality risk factors among different treatment groups.

### 2.4 Statistical analysis

We conducted the analysis using SPSS software for windows (version 26.0). We conducted univariate and multivariate analyses using the Kaplan-Meyer test and the Cox regression test. These analyses were conducted according to a separate comparison for each of surgery, chemotherapy, radiation, and radiation sequence with surgery. We presented the data as median (months) and 95% confidence interval (CI), and hazard ratio and 95% CI data of the univariate and multivariate analyses, respectively. We considered any analysis to be significant when the P-value was less than 0.05.

## 3 Results

### 3.1 Characteristics of the study population

We reviewed data of 68270 hepatocellular carcinoma patients, 56347 of them were dead. The mean age was 59.88 for the living patients and 63.27 for the dead, with a total mean age of 62.68 years. The majority of all patients were males (52342; 76.7%), and were White (45137; 66.1%). 37407 (54.8%) of the patients were married, 34064 (49.9%) were diagnosed at a localized disease stage, 10070 (14.8%) had grade II, and 43969 (64.4%) had an unknown grade. 24258 (35.5%) and 16456 (24.1%) received chemotherapy and underwent surgery, respectively. On the other hand, 63763 (93.4%) patients did not receive radiation therapy, and 67673 (99.1%) had no radiation therapy or surgery. Table 1 shows the details of the study population's characteristics.

**Table 1:** Baseline features of hepatocellular carcinoma patients and comparison according to vital status.

Variables	Alive (11923)	Dead (56347)	Total (68270)
Age	59.88 (10.545)	63.27 (11.661)	62.68 (11.545)
Sex			
Male	8896 (74.6%)	43446 (77.1%)	52342 (76.7%)
Female	3027 (25.4%)	12901 (22.9%)	15928 (23.3%)
Race			
White	7725 (64.8%)	37412 (66.4%)	45137 (66.1%)
Black	1214 (10.2%)	7931 (14.1%)	9145 (13.4%)
Others	2984 (25%)	11004 (19.5%)	13988 (20.5%)
Marital status			
Married	7268 (61%)	30139 (53.5%)	37407 (54.8%)
Single	2344 (19.7%)	11706 (20.8%)	14050 (20.6%)
Separated	210 (1.8%)	998 (1.8%)	1208 (1.8%)
Divorced	1354 (11.4%)	7090 (12.6%)	8444 (12.4%)
Widowed	747 (6.3%)	6414 (11.4%)	7161 (10.5%)
Stage			
Localized	9339 (78.3%)	24725 (43.9%)	34064 (49.9%)
Regional	2209 (18.5%)	19348 (34.3%)	21557 (31.6%)
Distant	375 (3.1%)	12274 (21.8%)	12649 (18.5%)
Grade			
I	1923 (16.1%)	6070 (10.8%)	7993 (11.7%)
II	2687 (22.5%)	7383 (13.1%)	10070 (14.8%)
III	754 (6.3%)	4858 (8.6%)	5612 (8.2%)
IV	61 (0.5%)	565 (1%)	626 (0.9%)
Unknown	6498 (54.5%)	37471 (66.5%)	43969 (64.4%)
Chemotherapy			
Yes	5332 (44.7%)	18926 (33.6%)	24258 (35.5%)
No	6591 (55.3%)	37421 (66.4%)	44012 (64.5%)
Surgery			
Yes	6728 (56.4%)	9728 (17.3%)	16456 (24.1%)
No	5195 (43.6%)	46619 (82.7%)	51814 (75.9%)
Radiation			
Beam	211 (1.8%)	2455 (4.4%)	2666 (3.9%)
Others	503 (4.2%)	1338 (2.4%)	1841 (2.7%)
None	11209 (94%)	52554 (93.3%)	63763 (93.4%)
Radiation sequence with surgery			
After surgery	49 (0.4%)	321 (0.6%)	370 (0.5%)
Intraoperative	9 (0.1%)	21 (0%)	30 (0%)
Prior to surgery	98 (0.8%)	89 (0.2%)	187 (0.3%)
No radiation or surgery	11763 (98.7%)	55910 (99.2%)	67673 (99.1%)
Radiation before and after	3 (0%)	5 (0%)	8 (0%)
Surgery before and after	1 (0%)	1 (0%)	2 (0%)

### 3.2 Univariate and multivariate analyses according to surgery performance comparison (Table 2)

#### 3.2.1 Univariate analysis of patients who performed surgery

The median overall survival was equal in males and females (20 months) and in White and Black races (20 months), but higher in other races (21 months). According to the marital status, the median overall survival was the highest in married patients (21 months), equal in separated and divorced (20 months), and the lowest in single and widowed (19 months). Staging-wise, the localized stage had the longest median survival (24 months), followed by the regional (15 months), and the least with the distant (7 months). The higher the grade, the shorter the median survival; grade I had a median survival of 28 months, grade II had 23 months median survival, grade III had 14 months median survival, and the shortest survival was with grade IV (12 months).

#### 3.2.2 Univariate analysis of patients who did not perform surgery

Generally, the median overall survival was markedly lower in patients who did not perform surgery than those who performed it. The highest median survival reached 6 months only, which occurred only with grade I patients and patients with a localized stage. On the other hand, the lowest median survival occurred with patients having a distant stage (1 month). The majority of the different variables had a median survival of 3 months.

#### 3.2.3 Multivariate analysis of patients who performed surgery

The mortality risk was significantly higher in patients aged ≥50 years (HR, 1.143; 95% CI, 1.074–1.217) than patients <50 years. The risk was significantly lower in females than in males (HR, 0.94; 95% CI, 0.897–0.985).

Races other than Black had a significantly lower risk than White patients (HR, 0.856; 95% CI, 0.815–0.899). The mortality risk was significantly higher in single patients (HR, 1.095; 95% CI, 1.035–1.158) and widowed patients (HR, 1.284; 95% CI, 1.192–1.384) than married patients. Other details are provided in Table 2.

**Table 2:** Univariate and multivariate analyses according to surgery performance comparison.

Variables	Yes				No			
	Univariate	P-value	Multivariate	Regression coefficient	Univariate	P-value	Multivariate	Regression coefficient
Age		0.687				<0.001		
<50 years	18 (16.347–19.653)		Reference		3 (2.842–3.158)		Reference	
≥50 years	20 (19.352–20.648)		1.143 (1.074–1.217)^b^	0.134	3 (2.92–3.08)		1.019 (0.988–1.051)	0.019
Sex		0.043				0.006		
Male	20 (19.306–20.694)		Reference		3 (2.929–3.071)		Reference	
Female	20 (18.768–21.232)		0.94 (0.897–0.985)^a^	−0.062	3 (2.834–3.166)		0.952 (0.931–0.975)^b^	−0.049
Race		0.002				<0.001		
White	20 (19.27–20.73)		Reference		3 (2.906–3.094)		Reference	
Black	20 (18.333–21.667)		1.057 (0.991–1.128)	0.056	3 (2.841–3.159)		1.053 (1.025–1.081)^b^	0.051
Others	21 (19.598–22.402)		0.856 (0.815–0.899)^b^	−0.155	3 (2.829–3.171)		0.916 (0.894–0.938)^b^	−0.088
Marital status		<0.001				<0.001		
Married	21 (20.208–21.792)		Reference		3 (2.899–3.101)		Reference	
Single	19 (17.444–20.556)		1.095 (1.035–1.158)^a^	0.091	3 (2.856–3.144)		1.064 (1.039–1.09)^b^	0.062
Separated	20 (16.433–23.567)		1.05 (0.901–1.225)	0.049	3 (2.277–3.723)		1.009 (0.942–1.082)	0.009
Divorced	20 (18.345–21.655)		1.061 (0.995–1.132)	0.059	4 (3.764–4.236)		1.025 (0.996–1.055)	0.025
Widowed	19 (17.211–20.789)		1.284 (1.192–1.384)^b^	0.25	3 (2.842–3.158)		1.226 (1.189–1.264)^b^	0.204
Stage		<0.001				<0.001		
Localized	24 (23.197–24.803)		Reference		6 (5.787–6.213)		Reference	
Regional	15 (14.068–15.932)		1.341 (1.279–1.407)^b^	0.294	3 (2.903–3.097)		1.425 (1.394–1.455)^b^	0.354
Distant	7 (6.088–7.912)		2.291 (2.109–2.487)^b^	0.829	1 (0.942–1.058)		2.089 (2.038–2.14)^b^	0.737
Grade		<0.001				<0.001		
I	28 (26.084–29.916)		Reference		6 (5.61–6.39)		Reference	
II	23 (21.752–24.248)		1.125 (1.059–1.195)^b^	0.118	4 (3.761–4.239)		1.206 (1.157–1.257)^b^	0.187
III	14 (12.554–15.446)		1.711 (1.589–1.842)^b^	0.537	2 (1.872–2.128)		1.741 (1.666–1.82)^b^	0.555
IV	12 (9.175–14.825)		1.894 (1.588–2.26)^b^	0.639	2 (1.713–2.287)		1.886 (1.708–2.082)^b^	0.634

a P < 0.05.

b P < 0.001.

#### 3.2.4 Multivariate analysis of patients who did not perform surgery

The mortality risk was significantly lower in females compared to males (HR, 0.952; 95% CI, 0.931–0.975). The death risk was significantly higher in Black, (HR, 1.053; 95% CI, 1.025–1.081) and significantly lower in races other than black (HR, 0.916; 95% CI, 0.894–0.938) compared to White races. The mortality risk was also significantly higher in single patients (HR, 1.064; 95% CI, 1.039–1.09) and widowed patients (HR, 1.226; 95% CI, 1.189–1.264), compared to married patients. Other details are provided in Table 2.

### 3.3 Univariate and multivariate analyses according to chemotherapy comparison (Table 3)

#### 3.3.1 Univariate analysis of patients who received chemotherapy

Patients with ≥50 years lived longer than patients <50 years (median 10 months and 8 months respectively). Females survived longer than males (median 11 months VS 10 months). White and races other than Black survived for a median of 10 months, while Black survived for a median of 9 months higher in other races (21 months). Other details are provided in Table 3.

**Table 3:** Univariate and multivariate analyses according to chemotherapy comparison.

Variables	Yes				No			
	Univariate	P-value	Multivariate	Regression coefficient	Univariate	P-value	Multivariate	Regression coefficient
Age		<0.001				0.426		
<50 years	8 (7.429–8.571)		Reference		2 (1.792–2.208)		Reference	
≥50 years	10 (9.759–10.241)		1.012 (0.966–1.061)	0.012	2 (1.929–2.071)		1.119 (1.081–1.159)^b^	0.113
Sex		<0.001				<0.001		
Male	10 (9.748–10.252)		Reference		2 (1.923–2.077)		Reference	
Female	11 (10.499–11.501)		0.948 (0.914–0.983)^a^	−0.054	3 (2.86–3.14)		0.917 (0.895–0.941)^b^	−0.086
Race		<0.001				<0.001		
White	10 (9.727–10.273)		Reference		2 (1.917–2.083)		Reference	
Black	9 (8.448–9.552)		1.081 (1.035–1.129)	0.078	2 (1.836–2.164)		1.049 (1.018–1.08)^a^	0.047
Others	10 (9.471–10.529)		0.908 (0.875–0.941)	−0.097	3 (2.843–3.157)		0.887 (0.864–0.911)^b^	−0.12
Marital status		0.338				<0.001		
Married	10 (9.703–10.297)		Reference		3 (2.905–3.095)		Reference	
Single	10 (9.501–10.499)		1 (0.962–1.04)	0	2 (1.894–2.106)		1.132 (1.102–1.163)^b^	0.124
Separated	11 (9.52–12.48)		1.076 (0.966–1.198)	0.073	3 (2.44–3.56)		1.035 (0.957–1.119)	0.034
Divorced	11 (10.398–11.602)		1.036 (0.991–1.084)	0.036	2 (1.814–2.186)		1.065 (1.032–1.1)^b^	0.063
Widowed	10 (9.275–10.725)		1.143 (1.081–1.208)^b^	0.134	2 (1.825–2.175)		1.267 (1.225–1.31)^b^	0.236
Stage		<0.001				<0.001		
Localized	15 (14.587–15.413)				6 (5.723–6.277)		Reference	
Regional	9 (8.696–9.304)		1.479 (1.432–1.527)^b^	0.391	2 (1.936–2.064)		1.67 (1.63–1.711)^b^	0.513
Distant	4 (3.784–4.216)		2.439 (2.34–2.541)^b^	0.891	1 (0.951–1.049)		2.398 (2.332–2.466)^b^	0.875
Grade		<0.001				<0.001		
I	14 (13.13–14.87)		Reference		7 (6.381–7.619)		Reference	
II	11 (10.343–11.657)		1.03 (0.971–1.093)	0.03	6 (5.482–6.518)		0.988 (0.948–1.03)	−0.012
III	6 (5.465–6.535)		1.741 (1.629–1.861)^b^	0.555	2 (1.883–2.117)		1.696 (1.62–1.776)^b^	0.528
IV	6 (4.834–7.166)		1.688 (1.446–1.969)^b^	0.523	2 (1.68–2.32)		2.022 (1.822–2.244)^b^	0.704

a P < 0.05.

b P < 0.001.

#### 3.3.2 Univariate analysis of patients who did not receive chemotherapy

Generally, the median overall survival was also noticeably lower in patients who did not receive chemotherapy than those who received it. Other details are provided in Table 3.

#### 3.3.3 Multivariate analysis of patients who received chemotherapy

The mortality risk was significantly lower in females than in males (HR, 0.948; 95% CI, 0.914–0.983), while significantly higher in widowed patients (HR, 1.143; 95% CI, 1.081–1.208) compared to married patients. Other details are provided in Table 3.

#### 3.3.4 Multivariate analysis of patients who did not receive chemotherapy

The death risk was significantly higher in patients aged ≥50 years (HR, 1.119; 95% CI, 1.081–1.159), compared to patients <50 years, but significantly lower in females compared to males (HR, 0.917; 95% CI, 0.895–0.941). The death risk was significantly higher in Black, (HR, 1.049; 95% CI, 1.018–1.08) and significantly lower in races other than black (HR, 0.887; 95% CI, 0.864–0.911) compared to White races. The mortality risk was also significantly higher in single patients (HR, 1.132; 95% CI, 1.102–1.163), divorced (HR, 1.065; 95% CI, 1.032–1.1), and widowed patients (HR, 1.267; 95% CI 1.225–1.31), compared to married patients. Other details are provided in Table 3.

### 3.4 Univariate and multivariate analyses according to radiation comparison (Table 4)

#### 3.4.1 Univariate analysis of patients who received beam

Patients with ≥50 years survived longer than patients <50 years (median 6 months and 4 months respectively), also females survived longer than males (median 6 months and 5 months respectively). White races lived for a median of 6 months, while Black and other races survived for a median of 5 months. Other details are provided in Table 4.

**Table 4:** Univariate and multivariate analyses according to radiation comparison.

Variables	Beam				Others				No			
	Univariate	P-value	Multivariate	Regression coefficient	Univariate	P-value	Multivariate	Regression coefficient	Univariate	P-value	Multivariate	Regression coefficient
Age		0.011				0.773				0.925		
<50 years	4 (3.275–4.725)		Reference		4 (3.718–4.282)		Reference		9 (6.58–11.42)		Reference	
≥50 years	6 (5.651–6.349)		0.983 (0.867–1.114)	−0.017	4 (3.898–4.102)		0.911 (0.726–1.143)^a^	−0.093	10 (9.428–10.572)		1.108 (1.077–1.141)^b^	0.103
Sex		0.009				0.066				<0.001		
Male	5 (4.654–5.346)		Reference		4 (3.899–4.101)		Reference		10 (9.403–10.597)		Reference	
Female	6 (5.099–6.901)		0.972 (0.874–1.082)	−0.028	5 (4.769–5.231)		0.903 (0.785–1.039)	−0.102	10 (8.508–11.492)		0.924 (0.904–0.944)^b^	−0.079
Race		<0.001				0.024				<0.001		
White	6 (5.545–6.455)		Reference		4 (3.88–4.12)		Reference		10 (9.326–10.674)		Reference	
Black	5 (4.318–5.682)		1.195 (1.06–1.347)^a^	0.178	3 (2.797–3.203)		1.121 (0.956–1.314)	0.114	9 (7.743–10.257)		1.059 (1.033–1.086)^b^	0.058
Others	5 (4.375–5.625)		1.103 (0.99–1.229)	0.098	5 (4.753–5.247)		0.823 (0.703–0.963)^a^	−0.195	12 (10.132–13.868)		0.879 (0.859–0.898)^b^	−0.129
Marital status		0.503				0.189				<0.001		
Married	5 (4.595–5.405)		Reference		5 (4.851–5.149)		Reference		10 (9.318–10.682)		Reference	
Single	5 (4.347–5.653)		1 (0.897–1.116)	0	4 (3.8–4.2)		0.959 (0.824–1.116)	−0.042	11 (9.382–12.618)		1.121 (1.095–1.146)^b^	0.114
Separated	6 (4.312–7.688)		1.06 (0.763–1.471)	0.058	5 (4.117–5.883)		1.32 (0.852–2.046)	0.278	7 (5.206–8.794)		1.049 (0.983–1.12)	0.048
Divorced	6 (4.869–7.131)		0.988 (0.872–1.12)	−0.012	5 (4.7–5.3)		1.059 (0.896–1.252)	0.058	10 (8.872–11.128)		1.072 (1.044–1.102)^b^	0.07
Widowed	5 (3.827–6.173)		1.181 (1.019–1.369)^a^	0.166	3 (2.809–3.191)		0.989 (0.803–1.219)	−0.011	10 (7.091–12.909)		1.312 (1.274–1.351)^b^	0.271
Stage		<0.001				<0.001				<0.001		
Localized	14 (12.683–15.317)		Reference		9 (8.722–9.278)		Reference		13 (11.902–14.098)		Reference	
Regional	7 (5.862–8.138)		1.439 (1.261–1.643)^b^	0.364	3 (2.883–3.117)		1.388 (1.235–1.561)^b^	0.328	9 (8.366–9.634)		1.582 (1.551–1.613)^b^	0.458
Distant	4 (3.728–4.272)		2.287 (2.046–2.557)^b^	0.827	1 (0.939–1.061)		1.786 (1.48–2.156)^b^	0.58	8 (6.606–9.394)		2.573 (2.511–2.637)^b^	0.945
Grade		<0.001				0.016				<0.001		
I	9 (7.166–10.834)		Reference		12 (9.827–14.173)		Reference		10 (9.435–10.565)		Reference	
II	7 (5.897–8.103)		1.129 (0.946–1.349)	0.122	12 (10.463–13.537)		1.102 (0.89–1.366)	0.098	8 (7.559–8.441)		0.996 (0.961–1.031)	−0.004
III	4 (3.26–4.74)		1.594 (1.335–1.903)^b^	0.466	8 (6.643–9.357)		1.71 (1.321–2.214)^b^	0.536	3 (2.807–3.193)		1.696 (1.631–1.764)^b^	0.528
IV	4 (1.456–6.544)		1.694 (1.138–2.524)^a^	0.527	9 (4.999–13.001)		1.879 (0.831–4.251)	0.631	2 (1.565–2.435)		1.899 (1.737–2.076)^b^	0.641

a P < 0.05.

b P < 0.001.

**Table 5:** Univariate and multivariate analyses according to radiation sequence with surgery comparison.

Variables	After surgery				Prior to surgery			
	Univariate	P-value	Multivariate	Regression coefficient	Univariate	P-value	Multivariate	Regression coefficient
Age		0.249				0.023		
<50 years	9 (5.538–12.462)		Reference		6 (2.151–9.849)		Reference	
≥50 years	12 (10.21–13.79)		1.05 (0.763–1.445)	0.049	15 (11.307–18.693)		0.321 (0.133–0.773)	−1.136
Sex		0.125				0.47		
Male	11 (9.314–12.686)		Reference		13 (9.304–16.696)		Reference	
Female	15 (10.56–19.44)		0.916 (0.688–1.219)	−0.088	14 (3.244–24.756)		1.308 (0.749–2.281)	0.268
Race		0.085				0.25		
White	12 (9.972–14.028)		Reference		14 (11.178–16.822)		Reference	
Black	11 (5.632–16.368)		1.265 (0.839–1.909)	0.235	9 (3.319–14.681)		1.603 (0.854–3.008)	0.472
Others	8 (6.28–9.72)		1.361 (0.992–1.867)	0.308	20 (0–42.722)		0.861 (0.472–1.569)	−0.15
Marital status		0.526				0.074		
Married	12 (9.603–14.397)		Reference		15 (10.458–19.542)		Reference	
Single	10 (6.614–13.386)		1.082 (0.799–1.464)	0.079	12 (2.2–21.8)		1.402 (0.748–2.63)	0.338
Separated	4 (0–14.78)		2.066 (0.722–5.916)	0.726	8 (5.228–10.772)		–	−10.456
Divorced	14 (4.938–23.062)		0.985 (0.689–1.406)	−0.016	13 (0–30.531)		2.507 (1.158–5.428)^a^	0.919
Widowed	8 (0–21.72)		1.294 (0.77–2.176)	0.258	14 (11.227–16.773)		0.792 (0.366–1.716)	−0.233
Stage		<0.001				<0.001		
Localized	20 (17.738–22.262)		Reference		22 (14.291–29.709)		Reference	
Regional	15 (11.168–18.832)		1.122 (0.812–1.551)	0.115	18 (0–39.329)		1.202 (0.679–2.127)	0.184
Distant	6 (4.914–7.086)		2.446 (1.851–3.232)^b^	0.894	7 (4.819–9.181)		4.415 (2.394–8.143)^b^	1.485
Grade		0.028				0.592		
I	18 (10.944–25.056)		Reference		14 (7.763–20.237)		Reference	
II	15 (10.715–19.285)		1.167 (0.773–1.761)	0.154	17 (9.174–24.826)		1.056 (0.564–1.977)	0.054
III	9 (6.814–11.186)		2.076 (1.336–3.227)^a^	0.731	15 (9.156–20.844)		0.959 (0.426–2.156)	−0.042
IV	10 (7.434–12.566)		2.48 (1.097–5.608)^a^	0.908	–		–	–

a P < 0.05.

b P < 0.001.

#### 3.4.2 Univariate analysis of patients who received others

Females survived longer than males (median 5 months and 4 months respectively). Races other than Black and White had a longer survival (median 5 months) than White (median 4 months) and Black races (median 3 months). Other details are provided in Table 4.

#### 3.4.3 Univariate analysis of patients did not receive radiation

Males and females had the same median survival (10 months). White races lived longer than Black (median 10 and 9 months respectively). However, other races had the longest median survival (12 months). Single patients had the longest median survival (11 months), and separated patients had the least (7 months). The localized stage had a median survival of 13 months, while the distant had 8 months. Lastly, grade I lived for a median of 10 months, while grade IV lived for a median of 2 months.

#### 3.4.4 Multivariate analysis of patients who received beam

The mortality risk was significantly higher in Black races than White (HR, 1.195; 95% CI, 1.06–1.347) and significantly higher in widowed patients (HR, 1.181; 95% CI, 1.019–1.369) compared to married patients. Other details are provided in Table 4.

#### 3.4.5 Multivariate analysis of patients who received others

The death risk was significantly lower in patients aged ≥50 years (HR, 0.911; 95% CI, 0.726–1.143) than patients <50 years. The mortality risk was significantly lower in races other than black (HR, 0.823; 95% CI, 0.703–0.963) compared to White races. Other details are provided in Table 4.

#### 3.4.6 Multivariate analysis of patients did not receive radiation

The death risk was significantly higher in patients aged ≥50 years (HR, 1.108; 95% CI, 1.077–1.141), compared to patients <50 years, but significantly lower in females than males (HR, 0.924; 95% CI, 0.904–0.944). The mortality risk was significantly higher in Black, but lower in races other than Black (HR, 1.059; 95% CI, 1.033–1.086) and (HR, 0.879; 95% CI, 0.859–0.898) respectively, compared to White races. Also, the death risk was significantly higher in single, divorced, and widowed patients compared to married ones (HR, 1.121; 95% CI, 1.095–1.146), (HR, 1.072; 95% CI, 1.044–1.102) and (HR, 1.312; 95% CI, 1.274–1.351) respectively. Other details are provided in Table 4.

### 3.5 Univariate and multivariate analyses according to radiation sequence with surgery comparison (Table 5)

#### 3.5.1 Univariate analysis of patients who received radiation after surgery

Patients with localized stage survived longer than other stages (median 20 months). Also, grade I patients survived longer than other grades (median 18 months).

#### 3.5.2 Univariate analysis of patients who received radiation prior to surgery

Patients aged ≥50 years survived longer than those <50 years (Median 15 and 6 months respectively), and patients with localized stage survived longer than other stages (median 22 months).

#### 3.5.3 Multivariate analysis of patients who received radiation after surgery

The death risk was significantly higher in patients with distant stage compared to the localized one (HR, 2.446; 95% CI, (1.851–3.232). Moreover, the risk was significantly higher in grades III and IV compared with grade I (HR, 2.076; 95% CI, (1.336–3.227) and (HR, 2.48; 95% CI, 1.097–5.608) respectively. Other variables showed no significant results.

#### 3.5.4 Mutlivariate analysis of patients who received radiation prior to surgery

The death risk was significantly higher in divorced compared with married patients (HR, 2.507; 95% CI, (1.158–5.428). Also, patients having a distant stage compared with the localized one (HR, 4.415; 95% CI, (2.394–8.143). Other variables had no significant results.

## 4 Discussion

The study showed that for patients aging ≥50 years, there was a higher mortality risk in those who performed surgery, did not receive chemotherapy, or did not receive radiation. However, they had a significantly lower mortality risk in cases receiving other radiation than the beam. Females had lower mortality risk in patients who performed surgery or not, received chemotherapy or not, or did not receive radiation. Patients with localized stage, or grade I had significantly lower mortality risks compared to their respective variable, whether they received any of our studied treatment options or not. Likely, married patients and other races also had a lower mortality risk than other marital statuses and White races. On the other hand, Blacks had a higher mortality risk than Whites.

In older people, choosing palliative treatment options could be due to co-existing medical morbidities. However, Shin et al. showed similar postoperative complications and overall survival in elderly and young patients (N = 233). They concluded that age alone should not be a determining HCC risk factor [14]. Another study also stated that elderly patients, with caution selection, had benefited from the major hepatectomy for large HCC as much as younger ones [15].

Black races had a higher mortality risk compared to White races. Generally, according to the racial/ethnic group, Black patients with HCC have the lowest overall survival [16,17]. This is not fully understood; however, it is probably multifactorial and may include socioeconomic factors and healthcare access variances [18,19]. Shaltiel et al. showed that at HCC diagnosis, in Black patients with a history of HCV infection, the liver fibrosis was significantly less advanced; however, their tumors were more advanced in stage and had worse pathologic prognostic features compared to non-Black patients [20]. Regarding the stages, similar to our results, several studies previously reported lower survival rates in regional and distant stages compared to the localized stage [21,22].

Similar to our results, a previous SEER study also showed an association between females and better survival in HCC patients, especially in younger cohorts. It is suggested that estrogen might protect against hepatocarcinogenesis and encourage more fortunate biology once HCC develops [23]. Contrary to this, Wu et al. did not report a survival difference between males and females [24].

Our study favored married patients. This could be explained by that, worldwide, being in a committed relationship is associated with a better lifestyle, including decreased smoking and alcohol ingestion, healthier diet, more physical activity, and maintenance of healthy body weight [[25], [26], [27], [28]]. Moreover, marriage can provide social support to relieve some stress and encourage keeping healthy lifestyle habits [29].

There is no enough data about the effect of radiation sequence with surgery in HCC, and this study mainly showed non-significant results. However, Wehling et al. recently stated that patients undergoing liver resection as an initial treatment had a median overall survival of 11.1 years, then those who underwent locoregional ablative intervention had 8.4 years. Initial transarterial chemoembolization treatment modality had 6.3 years median survival, whereas those treated with radiation had 2.9 or 5.5 years only [30].

We recommend future research to investigate other important variables and risk factors as well, such as hepatitis and obesity. We also encourage further investigation of the treatment sequence.

The strengths of this study include that the data are generalizable due to their high quality and precision, as they depend on the data registered in the SEER database. Moreover, the study had a large sample of HCC patients. We analyzed different variables with different treatment options. On the other hand, limitations of this study include the retrospective design and the missing of some important variables such as the environmental and genetic ones.

To conclude, in HCC patients who underwent surgery, those aged ≥50 years and grade II had higher mortality risks. Moreover, widowed, regional and distant stages, and grades III and IV had higher mortality risks whether with surgery, or chemotherapy, or radiation. Females had lower mortality risk in patients who performed surgery or not, received chemotherapy or not, or did not receive radiation. More investigations are needed to assess the radiation sequence with surgery.

## Funding

There are no funding sources for this work and no conflicts of interest and financial disclosures for the authors.

## Provenance and peer review

Not commissioned, externally peer-reviewed.

## Ethical approval

No Ethical Approval required.

## Consent

Not applicable.

## Author contribution

Kaif Qayum and Irfan Kar equally contributed to this paper and joined first authorship is proposed.Conception and design: Kaif Qayum, Irfan Kar Data collection: Kaif Qayum, Irfan Kar, Veena Sudarshan, Aliraza Syed Analysis and interpretation: Kaif Qayum, Irfan Kar Writing the article: Kaif Qayum, Irfan Kar, Ghulam Nawaz, Praveena Krishnakumar Critical revision of the article: All authors Final approval of the article: All authors Statistical analysis: Kaif Qayum, Irfan Kar.

## Registration of research studies

Researchregistry7087

https://www.researchregistry.com/browse-the-registry#home/registrationdetails/61235e24740bcc001e006ac8/

## Guarantor

Kaif Qayum.

## Declaration of competing interest

None.
